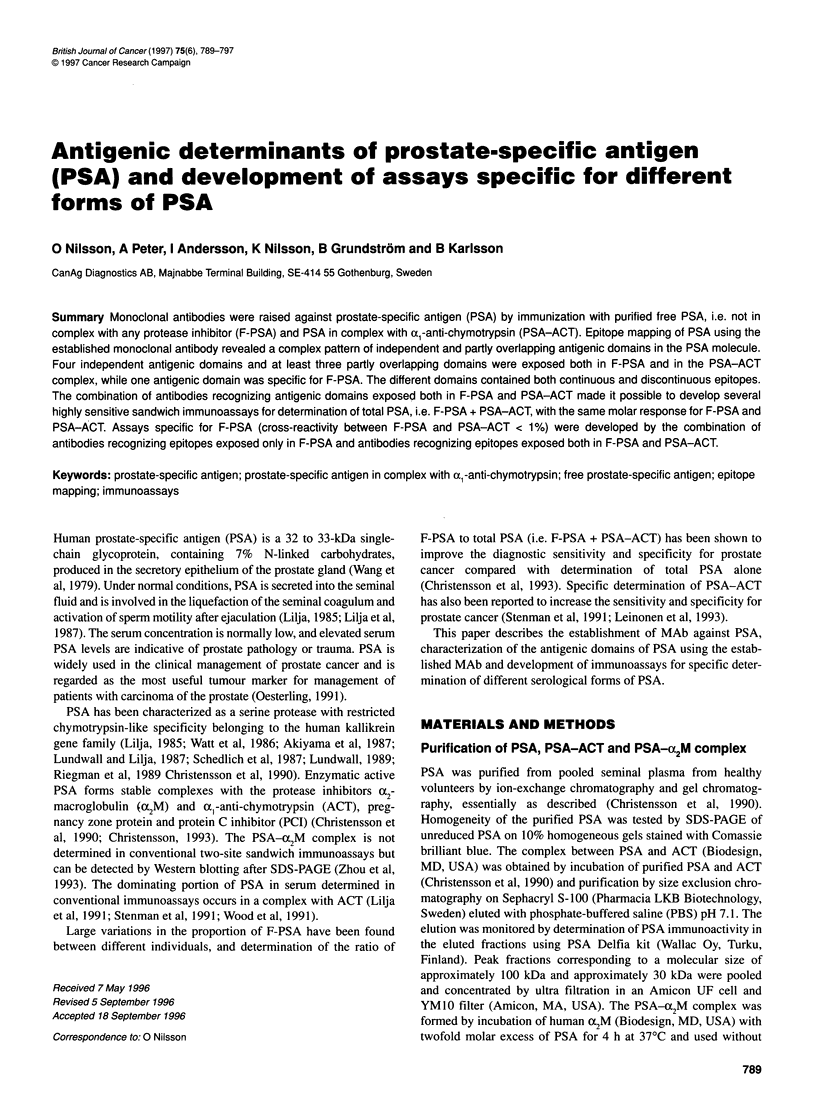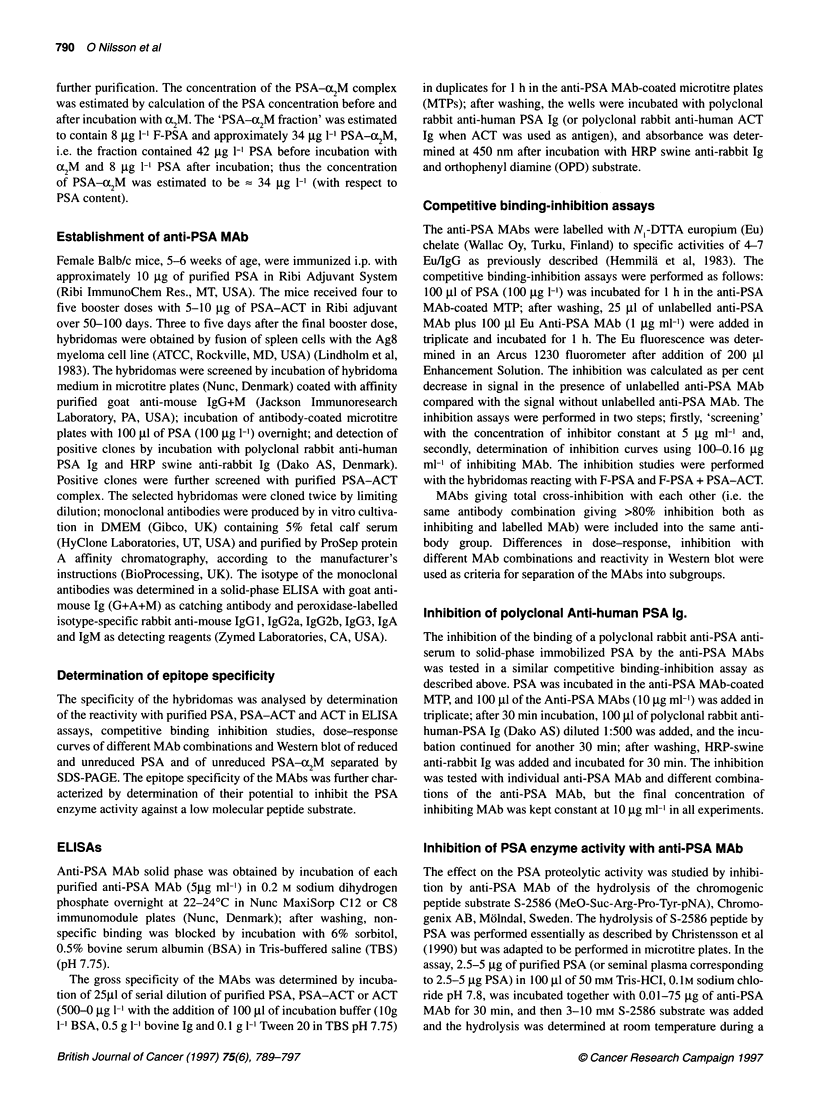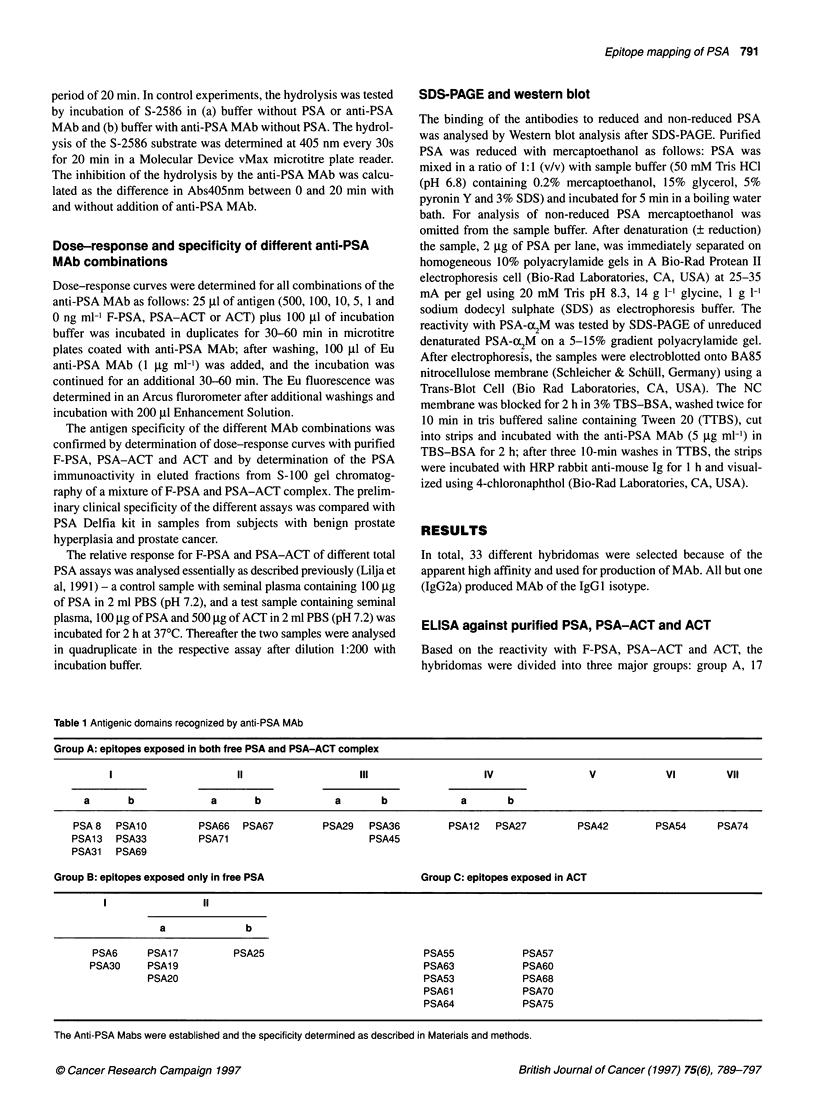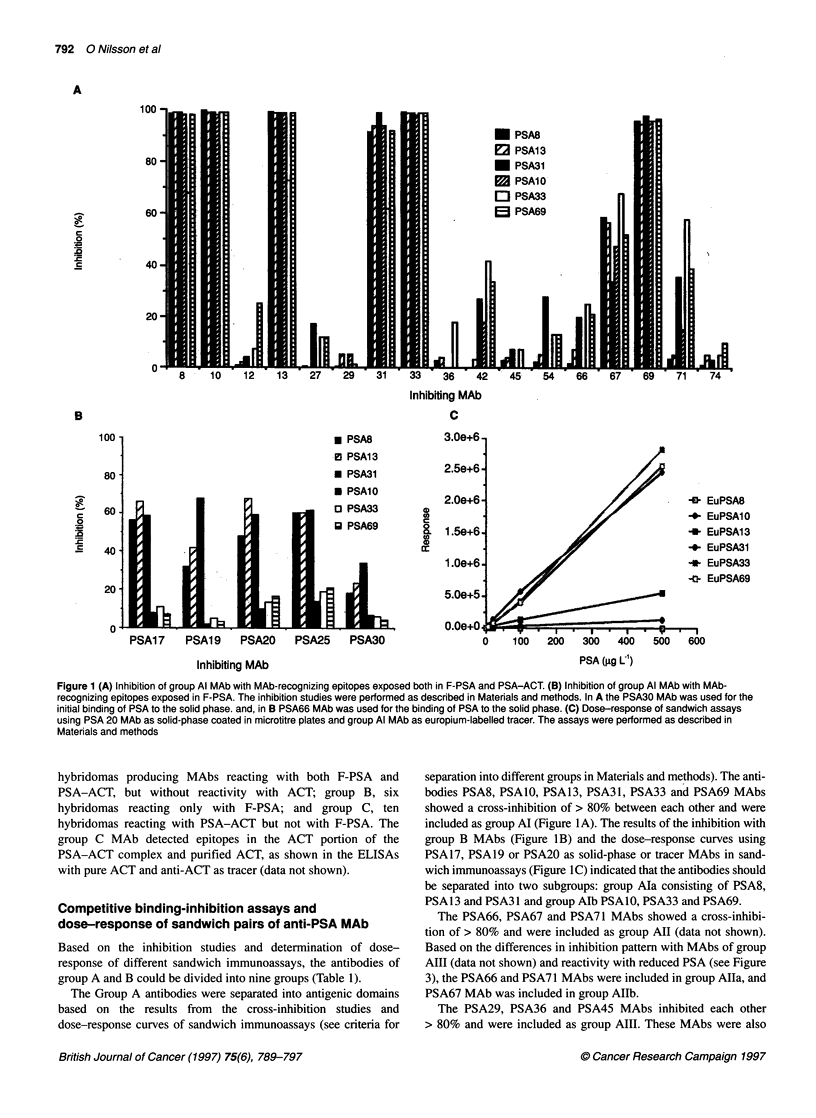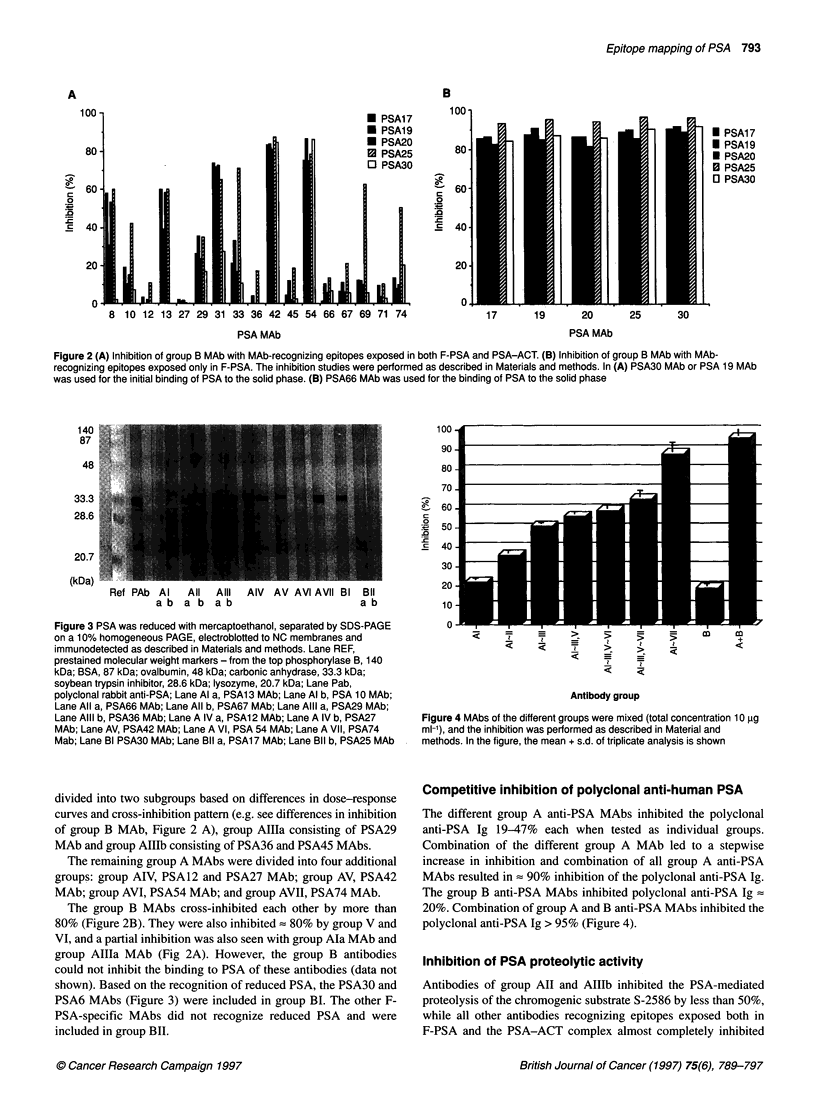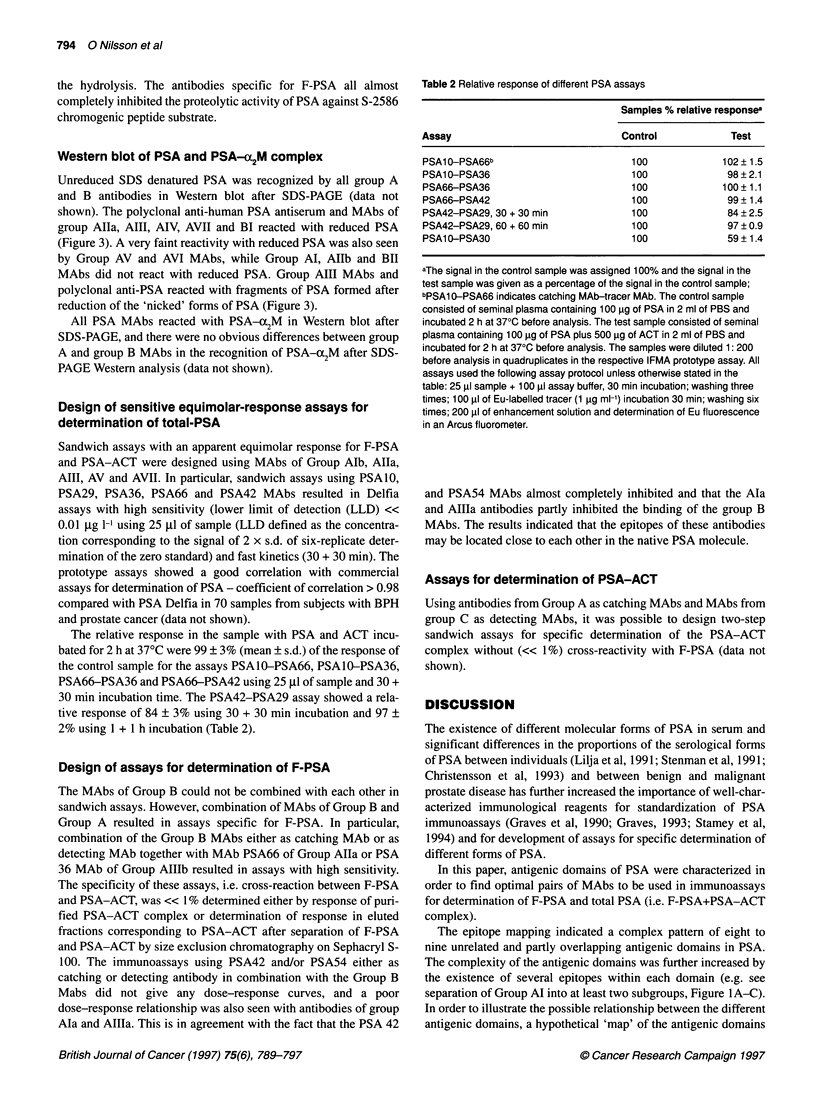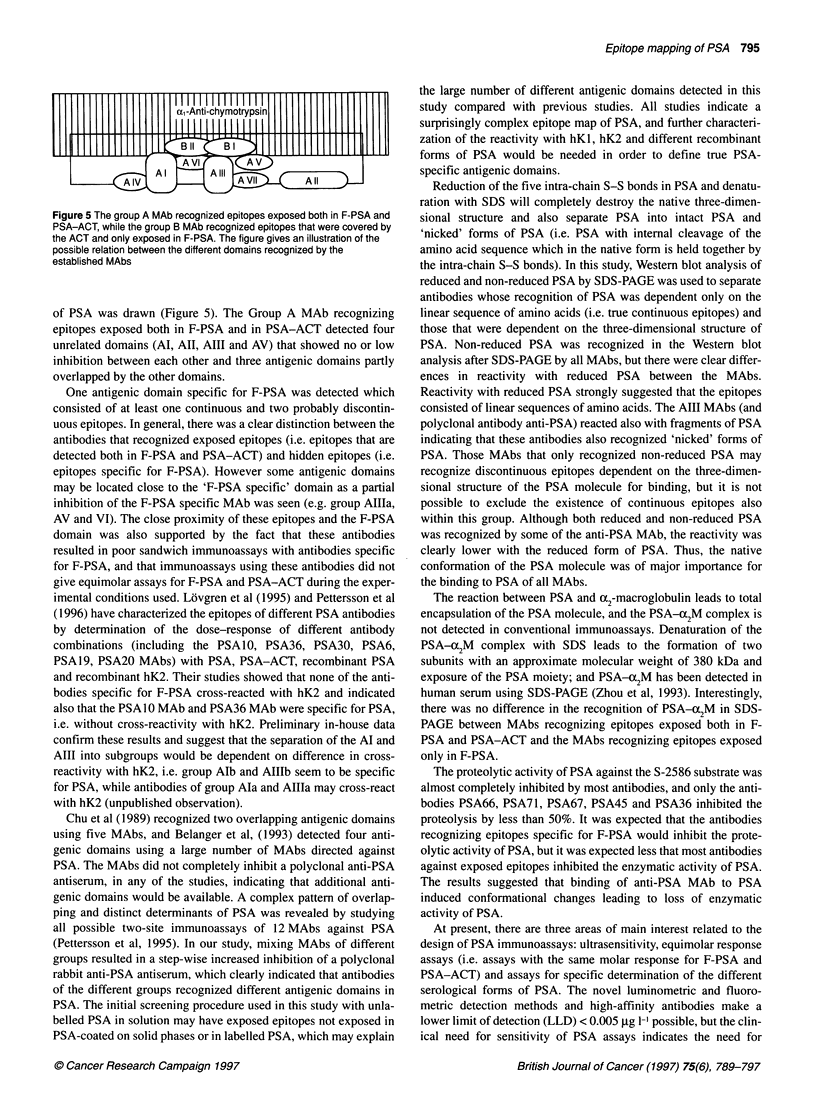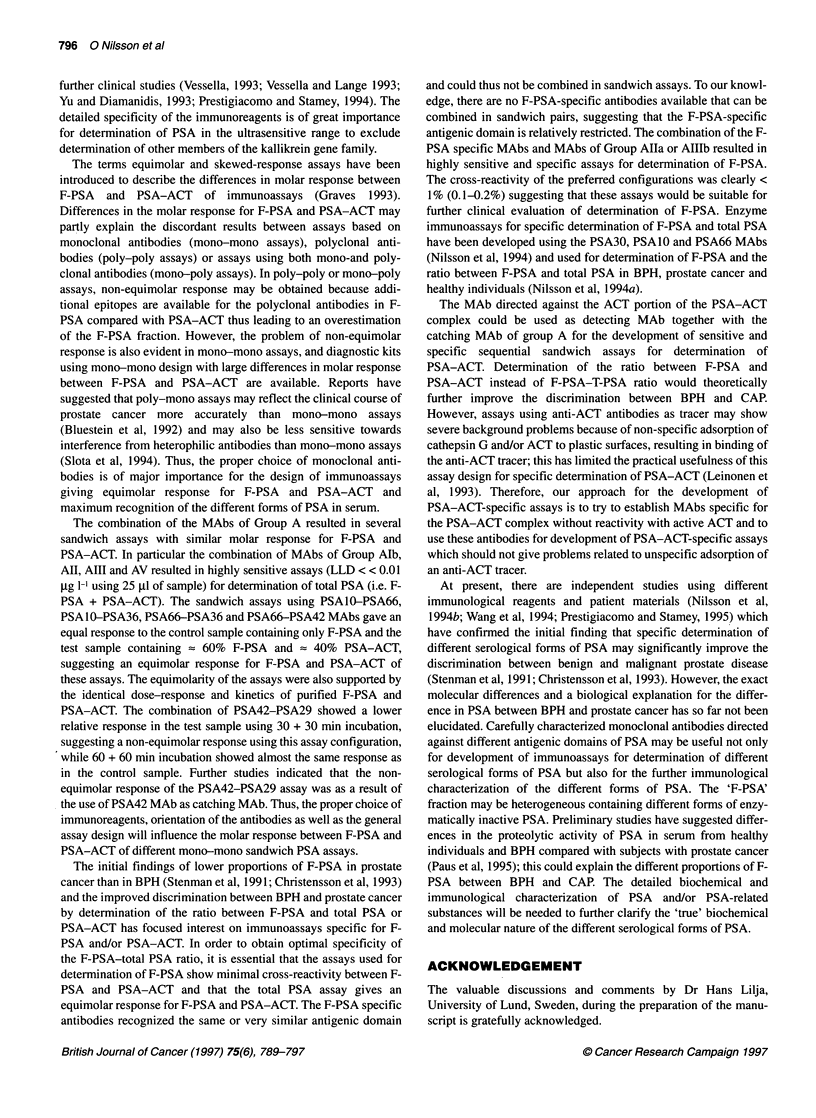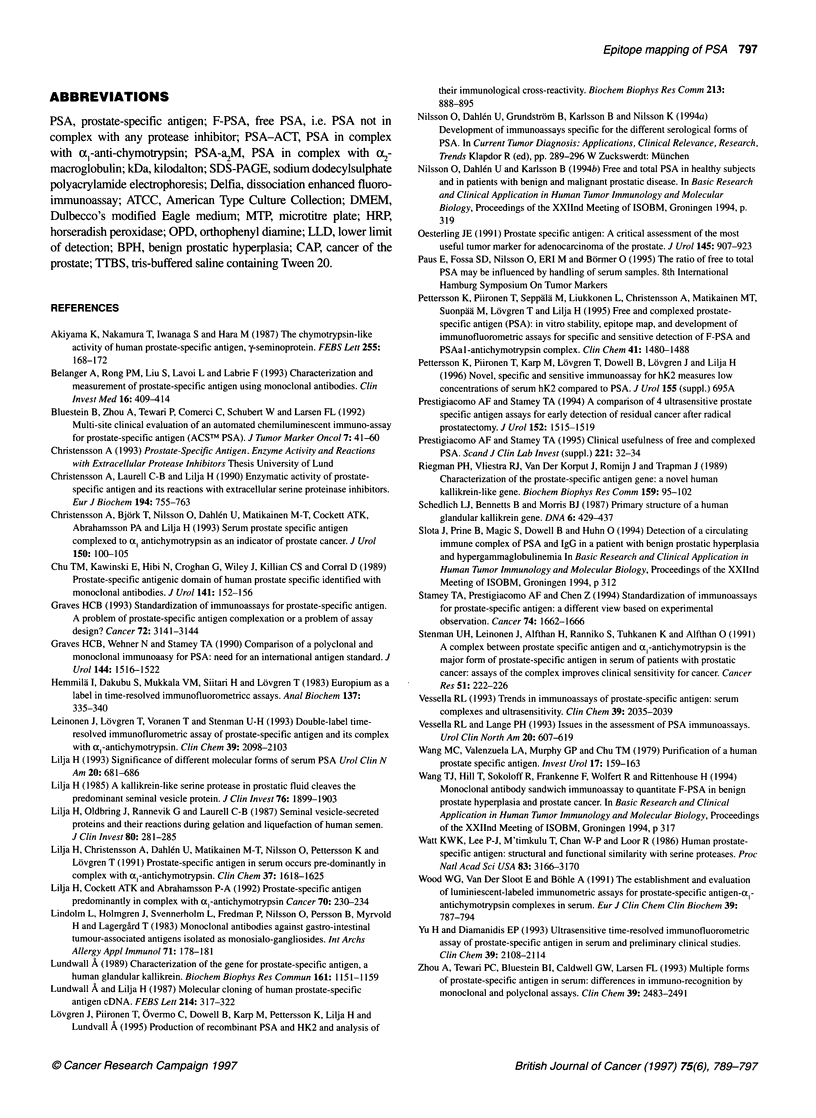# Antigenic determinants of prostate-specific antigen (PSA) and development of assays specific for different forms of PSA.

**DOI:** 10.1038/bjc.1997.142

**Published:** 1997

**Authors:** O. Nilsson, A. Peter, I. Andersson, K. Nilsson, B. Grundström, B. Karlsson

**Affiliations:** CanAg Diagnostics AB, Gothenburg, Sweden.

## Abstract

**Images:**


British Journal of Cancer (1997) 75(6), 789–797
© 1997 Cancer Research Campaign

### Antigenic determinants of prostate-specific antigen (PSA) and development of assays specific for different forms of PSA

**O Nilsson, A Peter, I Andersson, K Nilsson, B Grundström and B Karlsson**

CanAg Diagnostics AB, Majnabbe Terminal Building, SE-414 55 Gothenburg, Sweden

**Summary** Monoclonal antibodies were raised against prostate-specific antigen (PSA) by immunization with purified free PSA, i.e. not in complex with any protease inhibitor (F-PSA) and PSA in complex with $\alpha_1$-anti-chymotrypsin (PSA–ACT). Epitope mapping of PSA using the established monoclonal antibody revealed a complex pattern of independent and partly overlapping antigenic domains in the PSA molecule. Four independent antigenic domains and at least three partly overlapping domains were exposed both in F-PSA and in the PSA–ACT complex, while one antigenic domain was specific for F-PSA. The different domains contained both continuous and discontinuous epitopes. The combination of antibodies recognizing antigenic domains exposed both in F-PSA and PSA–ACT made it possible to develop several highly sensitive sandwich immunoassays for determination of total PSA, i.e. F-PSA + PSA–ACT, with the same molar response for F-PSA and PSA–ACT. Assays specific for F-PSA (cross-reactivity between F-PSA and PSA–ACT < 1%) were developed by the combination of antibodies recognizing epitopes exposed only in F-PSA and antibodies recognizing epitopes exposed both in F-PSA and PSA–ACT.

**Keywords:** prostate-specific antigen; prostate-specific antigen in complex with $\alpha_1$-anti-chymotrypsin; free prostate-specific antigen; epitope mapping; immunoassays

Human prostate-specific antigen (PSA) is a 32 to 33-kDa single-chain glycoprotein, containing 7% N-linked carbohydrates, produced in the secretory epithelium of the prostate gland (Wang et al, 1979). Under normal conditions, PSA is secreted into the seminal fluid and is involved in the liquefaction of the seminal coagulum and activation of sperm motility after ejaculation (Lilja, 1985; Lilja et al, 1987). The serum concentration is normally low, and elevated serum PSA levels are indicative of prostate pathology or trauma. PSA is widely used in the clinical management of prostate cancer and is regarded as the most useful tumour marker for management of patients with carcinoma of the prostate (Oesterling, 1991).

PSA has been characterized as a serine protease with restricted chymotrypsin-like specificity belonging to the human kallikrein gene family (Lilja, 1985; Watt et al, 1986; Akiyama et al, 1987; Lundwall and Lilja, 1987; Schedlich et al, 1987; Lundwall, 1989; Riegman et al, 1989 Christensson et al, 1990). Enzymatic active PSA forms stable complexes with the protease inhibitors $\alpha_2$-macroglobulin ($\alpha_2$M) and $\alpha_1$-anti-chymotrypsin (ACT), pregnancy zone protein and protein C inhibitor (PCI) (Christensson et al, 1990; Christensson, 1993). The PSA–$\alpha_2$M complex is not determined in conventional two-site sandwich immunoassays but can be detected by Western blotting after SDS-PAGE (Zhou et al, 1993). The dominating portion of PSA in serum determined in conventional immunoassays occurs in a complex with ACT (Lilja et al, 1991; Stenman et al, 1991; Wood et al, 1991).

Large variations in the proportion of F-PSA have been found between different individuals, and determination of the ratio of F-PSA to total PSA (i.e. F-PSA + PSA–ACT) has been shown to improve the diagnostic sensitivity and specificity for prostate cancer compared with determination of total PSA alone (Christensson et al, 1993). Specific determination of PSA–ACT has also been reported to increase the sensitivity and specificity for prostate cancer (Stenman et al, 1991; Leinonen et al, 1993).

This paper describes the establishment of MAb against PSA, characterization of the antigenic domains of PSA using the established MAb and development of immunoassays for specific determination of different serological forms of PSA.

*Received 7 May 1996*
*Revised 5 September 1996*
*Accepted 18 September 1996*

*Correspondence to:* O Nilsson

### MATERIALS AND METHODS

### Purification of PSA, PSA–ACT and PSA–$\alpha_2$M complex

PSA was purified from pooled seminal plasma from healthy volunteers by ion-exchange chromatography and gel chromatography, essentially as described (Christensson et al, 1990). Homogeneity of the purified PSA was tested by SDS-PAGE of unreduced PSA on 10% homogeneous gels stained with Comassie brilliant blue. The complex between PSA and ACT (Biodesign, MD, USA) was obtained by incubation of purified PSA and ACT (Christensson et al, 1990) and purification by size exclusion chromatography on Sephacryl S-100 (Pharmacia LKB Biotechnology, Sweden) eluted with phosphate-buffered saline (PBS) pH 7.1. The elution was monitored by determination of PSA immunoactivity in the eluted fractions using PSA Delfia kit (Wallac Oy, Turku, Finland). Peak fractions corresponding to a molecular size of approximately 100 kDa and approximately 30 kDa were pooled and concentrated by ultra filtration in an Amicon UF cell and YM10 filter (Amicon, MA, USA). The PSA–$\alpha_2$M complex was formed by incubation of human $\alpha_2$M (Biodesign, MD, USA) with twofold molar excess of PSA for 4 h at 37°C and used without further purification. The concentration of the PSA–$\alpha_2$M complex was estimated by calculation of the PSA concentration before and after incubation with $\alpha_2$M. The 'PSA–$\alpha_2$M fraction' was estimated to contain 8 μg $l^{-1}$ F-PSA and approximately 34 μg $l^{-1}$ PSA–$\alpha_2$M, i.e. the fraction contained 42 μg $l^{-1}$ PSA before incubation with $\alpha_2$M and 8 μg $l^{-1}$ PSA after incubation; thus the concentration of PSA–$\alpha_2$M was estimated to be ≈ 34 μg $l^{-1}$ (with respect to PSA content).

### Establishment of anti-PSA MAb

Female Balb/c mice, 5–6 weeks of age, were immunized i.p. with approximately 10 μg of purified PSA in Ribi Adjuvant System (Ribi ImmunoChem Res., MT, USA). The mice received four to five booster doses with 5–10 μg of PSA–ACT in Ribi adjuvant over 50–100 days. Three to five days after the final booster dose, hybridomas were obtained by fusion of spleen cells with the Ag8 myeloma cell line (ATCC, Rockville, MD, USA) (Lindholm et al, 1983). The hybridomas were screened by incubation of hybridoma medium in microtitre plates (Nunc, Denmark) coated with affinity purified goat anti-mouse IgG+M (Jackson Immunoresearch Laboratory, PA, USA); incubation of antibody-coated microtitre plates with 100 μl of PSA (100 μg $l^{-1}$) overnight; and detection of positive clones by incubation with polyclonal rabbit anti-human PSA Ig and HRP swine anti-rabbit Ig (Dako AS, Denmark). Positive clones were further screened with purified PSA–ACT complex. The selected hybridomas were cloned twice by limiting dilution; monoclonal antibodies were produced by in vitro cultivation in DMEM (Gibco, UK) containing 5% fetal calf serum (HyClone Laboratories, UT, USA) and purified by ProSep protein A affinity chromatography, according to the manufacturer's instructions (BioProcessing, UK). The isotype of the monoclonal antibodies was determined in a solid-phase ELISA with goat anti-mouse Ig (G+A+M) as catching antibody and peroxidase-labelled isotype-specific rabbit anti-mouse IgG1, IgG2a, IgG2b, IgG3, IgA and IgM as detecting reagents (Zymed Laboratories, CA, USA).

### Determination of epitope specificity

The specificity of the hybridomas was analysed by determination of the reactivity with purified PSA, PSA–ACT and ACT in ELISA assays, competitive binding inhibition studies, dose–response curves of different MAb combinations and Western blot of reduced and unreduced PSA and of unreduced PSA–$\alpha_2$M separated by SDS-PAGE. The epitope specificity of the MAbs was further characterized by determination of their potential to inhibit the PSA enzyme activity against a low molecular peptide substrate.

### ELISAs

Anti-PSA MAb solid phase was obtained by incubation of each purified anti-PSA MAb (5μg $ml^{-1}$) in 0.2 M sodium dihydrogen phosphate overnight at 22–24°C in Nunc MaxiSorp C12 or C8 immunomodule plates (Nunc, Denmark); after washing, non-specific binding was blocked by incubation with 6% sorbitol, 0.5% bovine serum albumin (BSA) in Tris-buffered saline (TBS) (pH 7.75).

The gross specificity of the MAbs was determined by incubation of 25μl of serial dilution of purified PSA, PSA–ACT or ACT (500–0 μg $l^{-1}$ with the addition of 100 μl of incubation buffer (10g $l^{-1}$ BSA, 0.5 g $l^{-1}$ bovine Ig and 0.1 g $l^{-1}$ Tween 20 in TBS pH 7.75) in duplicates for 1 h in the anti-PSA MAb-coated microtitre plates (MTPs); after washing, the wells were incubated with polyclonal rabbit anti-human PSA Ig (or polyclonal rabbit anti-human ACT Ig when ACT was used as antigen), and absorbance was determined at 450 nm after incubation with HRP swine anti-rabbit Ig and orthophenyl diamine (OPD) substrate.

### Competitive binding-inhibition assays

The anti-PSA MAbs were labelled with $N_1$-DTTA europium (Eu) chelate (Wallac Oy, Turku, Finland) to specific activities of 4–7 Eu/IgG as previously described (Hemmilä et al, 1983). The competitive binding-inhibition assays were performed as follows: 100 μl of PSA (100 μg $l^{-1}$) was incubated for 1 h in the anti-PSA MAb-coated MTP; after washing, 25 μl of unlabelled anti-PSA MAb plus 100 μl Eu Anti-PSA MAb (1 μg $ml^{-1}$) were added in triplicate and incubated for 1 h. The Eu fluorescence was determined in an Arcus 1230 fluorometer after addition of 200 μl Enhancement Solution. The inhibition was calculated as per cent decrease in signal in the presence of unlabelled anti-PSA MAb compared with the signal without unlabelled anti-PSA MAb. The inhibition assays were performed in two steps; firstly, 'screening' with the concentration of inhibitor constant at 5 μg $ml^{-1}$ and, secondly, determination of inhibition curves using 100–0.16 μg $ml^{-1}$ of inhibiting MAb. The inhibition studies were performed with the hybridomas reacting with F-PSA and F-PSA + PSA–ACT.

MAbs giving total cross-inhibition with each other (i.e. the same antibody combination giving >80% inhibition both as inhibiting and labelled MAb) were included into the same antibody group. Differences in dose–response, inhibition with different MAb combinations and reactivity in Western blot were used as criteria for separation of the MAbs into subgroups.

### Inhibition of polyclonal Anti-human PSA Ig.

The inhibition of the binding of a polyclonal rabbit anti-PSA antiserum to solid-phase immobilized PSA by the anti-PSA MAbs was tested in a similar competitive binding-inhibition assay as described above. PSA was incubated in the anti-PSA MAb-coated MTP, and 100 μl of the Anti-PSA MAbs (10 μg $ml^{-1}$) was added in triplicate; after 30 min incubation, 100 μl of polyclonal rabbit anti-human-PSA Ig (Dako AS) diluted 1:500 was added, and the incubation continued for another 30 min; after washing, HRP-swine anti-rabbit Ig was added and incubated for 30 min. The inhibition was tested with individual anti-PSA MAb and different combinations of the anti-PSA MAb, but the final concentration of inhibiting MAb was kept constant at 10 μg $ml^{-1}$ in all experiments.

### Inhibition of PSA enzyme activity with anti-PSA MAb

The effect on the PSA proteolytic activity was studied by inhibition by anti-PSA MAb of the hydrolysis of the chromogenic peptide substrate S-2586 (MeO-Suc-Arg-Pro-Tyr-pNA), Chromogenix AB, Mölndal, Sweden. The hydrolysis of S-2586 peptide by PSA was performed essentially as described by Christensson et al (1990) but was adapted to be performed in microtitre plates. In the assay, 2.5–5 μg of purified PSA (or seminal plasma corresponding to 2.5–5 μg PSA) in 100 μl of 50 mM Tris-HCl, 0.1M sodium chloride pH 7.8, was incubated together with 0.01–75 μg of anti-PSA MAb for 30 min, and then 3–10 mM S-2586 substrate was added and the hydrolysis was determined at room temperature during a

© Cancer Research Campaign 1997

period of 20 min. In control experiments, the hydrolysis was tested by incubation of S-2586 in (a) buffer without PSA or anti-PSA MAb and (b) buffer with anti-PSA MAb without PSA. The hydrolysis of the S-2586 substrate was determined at 405 nm every 30s for 20 min in a Molecular Device vMax microtitre plate reader. The inhibition of the hydrolysis by the anti-PSA MAb was calculated as the difference in Abs405nm between 0 and 20 min with and without addition of anti-PSA MAb.

### Dose–response and specificity of different anti-PSA MAb combinations

Dose–response curves were determined for all combinations of the anti-PSA MAb as follows: 25 µl of antigen (500, 100, 10, 5, 1 and 0 ng ml$^{-1}$ F-PSA, PSA–ACT or ACT) plus 100 µl of incubation buffer was incubated in duplicates for 30–60 min in microtitre plates coated with anti-PSA MAb; after washing, 100 µl of Eu anti-PSA MAb (1 µg ml$^{-1}$) was added, and the incubation was continued for an additional 30–60 min. The Eu fluorescence was determined in an Arcus flurorometer after additional washings and incubation with 200 µl Enhancement Solution.

The antigen specificity of the different MAb combinations was confirmed by determination of dose–response curves with purified F-PSA, PSA–ACT and ACT and by determination of the PSA immunoactivity in eluted fractions from S-100 gel chromatography of a mixture of F-PSA and PSA–ACT complex. The preliminary clinical specificity of the different assays was compared with PSA Delfia kit in samples from subjects with benign prostate hyperplasia and prostate cancer.

The relative response for F-PSA and PSA–ACT of different total PSA assays was analysed essentially as described previously (Lilja et al, 1991) – a control sample with seminal plasma containing 100 µg of PSA in 2 ml PBS (pH 7.2), and a test sample containing seminal plasma, 100 µg of PSA and 500 µg of ACT in 2 ml PBS (pH 7.2) was incubated for 2 h at 37°C. Thereafter the two samples were analysed in quadruplicate in the respective assay after dilution 1:200 with incubation buffer.

### SDS-PAGE and western blot

The binding of the antibodies to reduced and non-reduced PSA was analysed by Western blot analysis after SDS-PAGE. Purified PSA was reduced with mercaptoethanol as follows: PSA was mixed in a ratio of 1:1 (v/v) with sample buffer (50 mM Tris HCl (pH 6.8) containing 0.2% mercaptoethanol, 15% glycerol, 5% pyronin Y and 3% SDS) and incubated for 5 min in a boiling water bath. For analysis of non-reduced PSA mercaptoethanol was omitted from the sample buffer. After denaturation (± reduction) the sample, 2 µg of PSA per lane, was immediately separated on homogeneous 10% polyacrylamide gels in A Bio-Rad Protean II electrophoresis cell (Bio-Rad Laboratories, CA, USA) at 25–35 mA per gel using 20 mM Tris pH 8.3, 14 g l$^{-1}$ glycine, 1 g l$^{-1}$ sodium dodecyl sulphate (SDS) as electrophoresis buffer. The reactivity with PSA-$\alpha_2$M was tested by SDS-PAGE of unreduced denaturated PSA-$\alpha_2$M on a 5–15% gradient polyacrylamide gel. After electrophoresis, the samples were electroblotted onto BA85 nitrocellulose membrane (Schleicher & Schüll, Germany) using a Trans-Blot Cell (Bio Rad Laboratories, CA, USA). The NC membrane was blocked for 2 h in 3% TBS–BSA, washed twice for 10 min in tris buffered saline containing Tween 20 (TTBS), cut into strips and incubated with the anti-PSA MAb (5 µg ml$^{-1}$) in TBS–BSA for 2 h; after three 10-min washes in TTBS, the strips were incubated with HRP rabbit anti-mouse Ig for 1 h and visualized using 4-chloronaphthol (Bio-Rad Laboratories, CA, USA).

### RESULTS

In total, 33 different hybridomas were selected because of the apparent high affinity and used for production of MAb. All but one (IgG2a) produced MAb of the IgG1 isotype.

### ELISA against purified PSA, PSA–ACT and ACT

Based on the reactivity with F-PSA, PSA–ACT and ACT, the hybridomas were divided into three major groups: group A, 17

**Table 1** Antigenic domains recognized by anti-PSA MAb

**Group A: epitopes exposed in both free PSA and PSA–ACT complex**

| I | | II | | III | | IV | | V | VI | VII |
|---|---|---|---|---|---|---|---|---|---|---|
| a | b | a | b | a | b | a | b | | | |
| PSA 8<br>PSA13<br>PSA31 | PSA10<br>PSA33<br>PSA69 | PSA66<br>PSA71 | PSA67 | PSA29 | PSA36<br>PSA45 | PSA12 | PSA27 | PSA42 | PSA54 | PSA74 |

**Group B: epitopes exposed only in free PSA**

| I | II | |
|---|---|---|
| | a | b |
| PSA6<br>PSA30 | PSA17<br>PSA19<br>PSA20 | PSA25 |

**Group C: epitopes exposed in ACT**

| | |
|---|---|
| PSA55 | PSA57 |
| PSA63 | PSA60 |
| PSA53 | PSA68 |
| PSA61 | PSA70 |
| PSA64 | PSA75 |

The Anti-PSA Mabs were established and the specificity determined as described in Materials and methods.

© Cancer Research Campaign 1997

**Figure 1 (A)** Inhibition of group AI MAb with MAb-recognizing epitopes exposed both in F-PSA and PSA–ACT. **(B)** Inhibition of group AI MAb with MAb-recognizing epitopes exposed in F-PSA. The inhibition studies were performed as described in Materials and methods. In **A** the PSA30 MAb was used for the initial binding of PSA to the solid phase. and, in **B** PSA66 MAb was used for the binding of PSA to the solid phase. **(C)** Dose–response of sandwich assays using PSA 20 MAb as solid-phase coated in microtitre plates and group AI MAb as europium-labelled tracer. The assays were performed as described in Materials and methods hybridomas producing MAbs reacting with both F-PSA and PSA–ACT, but without reactivity with ACT; group B, six hybridomas reacting only with F-PSA; and group C, ten hybridomas reacting with PSA–ACT but not with F-PSA. The group C MAb detected epitopes in the ACT portion of the PSA–ACT complex and purified ACT, as shown in the ELISAs with pure ACT and anti-ACT as tracer (data not shown).

### Competitive binding-inhibition assays and dose–response of sandwich pairs of anti-PSA MAb

Based on the inhibition studies and determination of dose–response of different sandwich immunoassays, the antibodies of group A and B could be divided into nine groups (Table 1).

The Group A antibodies were separated into antigenic domains based on the results from the cross-inhibition studies and dose–response curves of sandwich immunoassays (see criteria for separation into different groups in Materials and methods). The antibodies PSA8, PSA10, PSA13, PSA31, PSA33 and PSA69 MAbs showed a cross-inhibition of > 80% between each other and were included as group AI (Figure 1A). The results of the inhibition with group B MAbs (Figure 1B) and the dose–response curves using PSA17, PSA19 or PSA20 as solid-phase or tracer MAbs in sandwich immunoassays (Figure 1C) indicated that the antibodies should be separated into two subgroups: group AIa consisting of PSA8, PSA13 and PSA31 and group AIb PSA10, PSA33 and PSA69.

The PSA66, PSA67 and PSA71 MAbs showed a cross-inhibition of > 80% and were included as group AII (data not shown). Based on the differences in inhibition pattern with MAbs of group AIII (data not shown) and reactivity with reduced PSA (see Figure 3), the PSA66 and PSA71 MAbs were included in group AIIa, and PSA67 MAb was included in group AIIb.

The PSA29, PSA36 and PSA45 MAbs inhibited each other > 80% and were included as group AIII. These MAbs were also

© Cancer Research Campaign 1997

**Figure 2 (A)** Inhibition of group B MAb with MAb-recognizing epitopes exposed in both F-PSA and PSA–ACT. **(B)** Inhibition of group B MAb with MAb-recognizing epitopes exposed only in F-PSA. The inhibition studies were performed as described in Materials and methods. In **(A)** PSA30 MAb or PSA 19 MAb was used for the initial binding of PSA to the solid phase. **(B)** PSA66 MAb was used for the binding of PSA to the solid phase

**Figure 3** PSA was reduced with mercaptoethanol, separated by SDS-PAGE on a 10% homogeneous PAGE, electroblotted to NC membranes and immunodetected as described in Materials and methods. Lane REF, prestained molecular weight markers – from the top phosphorylase B, 140 kDa; BSA, 87 kDa; ovalbumin, 48 kDa; carbonic anhydrase, 33.3 kDa; soybean trypsin inhibitor, 28.6 kDa; lysozyme, 20.7 kDa; Lane Pab, polyclonal rabbit anti-PSA; Lane AI a, PSA13 MAb; Lane AI b, PSA 10 MAb; Lane AII a, PSA66 MAb; Lane AII b, PSA67 MAb; Lane AIII a, PSA29 MAb; Lane AIII b, PSA36 MAb; Lane A IV a, PSA12 MAb; Lane A IV b, PSA27 MAb; Lane AV, PSA42 MAb; Lane A VI, PSA 54 MAb; Lane A VII, PSA74 Mab; Lane BI PSA30 MAb; Lane BII a, PSA17 MAb; Lane BII b, PSA25 MAb

**Figure 4** MAbs of the different groups were mixed (total concentration 10 µg ml$^{-1}$), and the inhibition was performed as described in Material and methods. In the figure, the mean + s.d. of triplicate analysis is shown divided into two subgroups based on differences in dose–response curves and cross-inhibition pattern (e.g. see differences in inhibition of group B MAb, Figure 2 A), group AIIIa consisting of PSA29 MAb and group AIIIb consisting of PSA36 and PSA45 MAbs.

The remaining group A MAbs were divided into four additional groups: group AIV, PSA12 and PSA27 MAb; group AV, PSA42 MAb; group AVI, PSA54 MAb; and group AVII, PSA74 MAb.

The group B MAbs cross-inhibited each other by more than 80% (Figure 2B). They were also inhibited ≈ 80% by group V and VI, and a partial inhibition was also seen with group AIa MAb and group AIIIa MAb (Fig 2A). However, the group B antibodies could not inhibit the binding to PSA of these antibodies (data not shown). Based on the recognition of reduced PSA, the PSA30 and PSA6 MAbs (Figure 3) were included in group BI. The other F-PSA-specific MAbs did not recognize reduced PSA and were included in group BII.

### Competitive inhibition of polyclonal anti-human PSA

The different group A anti-PSA MAbs inhibited the polyclonal anti-PSA Ig 19–47% each when tested as individual groups. Combination of the different group A MAb led to a stepwise increase in inhibition and combination of all group A anti-PSA MAbs resulted in ≈ 90% inhibition of the polyclonal anti-PSA Ig. The group B anti-PSA MAbs inhibited polyclonal anti-PSA Ig ≈ 20%. Combination of group A and B anti-PSA MAbs inhibited the polyclonal anti-PSA Ig > 95% (Figure 4).

### Inhibition of PSA proteolytic activity

Antibodies of group AII and AIIIb inhibited the PSA-mediated proteolysis of the chromogenic substrate S-2586 by less than 50%, while all other antibodies recognizing epitopes exposed both in F-PSA and the PSA–ACT complex almost completely inhibited

© Cancer Research Campaign 1997

the hydrolysis. The antibodies specific for F-PSA all almost completely inhibited the proteolytic activity of PSA against S-2586 chromogenic peptide substrate.

### Western blot of PSA and PSA–$\alpha_2$M complex

Unreduced SDS denatured PSA was recognized by all group A and B antibodies in Western blot after SDS-PAGE (data not shown). The polyclonal anti-human PSA antiserum and MAbs of group AIIa, AIII, AIV, AVII and BI reacted with reduced PSA (Figure 3). A very faint reactivity with reduced PSA was also seen by Group AV and AVI MAbs, while Group AI, AIIb and BII MAbs did not react with reduced PSA. Group AIII MAbs and polyclonal anti-PSA reacted with fragments of PSA formed after reduction of the 'nicked' forms of PSA (Figure 3).

All PSA MAbs reacted with PSA–$\alpha_2$M in Western blot after SDS-PAGE, and there were no obvious differences between group A and group B MAbs in the recognition of PSA–$\alpha_2$M after SDS-PAGE Western analysis (data not shown).

### Design of sensitive equimolar-response assays for determination of total-PSA

Sandwich assays with an apparent equimolar response for F-PSA and PSA–ACT were designed using MAbs of Group AIb, AIIa, AIII, AV and AVII. In particular, sandwich assays using PSA10, PSA29, PSA36, PSA66 and PSA42 MAbs resulted in Delfia assays with high sensitivity (lower limit of detection (LLD) << 0.01 μg $l^{-1}$ using 25 μl of sample (LLD defined as the concentration corresponding to the signal of 2 × s.d. of six-replicate determination of the zero standard) and fast kinetics (30 + 30 min). The prototype assays showed a good correlation with commercial assays for determination of PSA – coefficient of correlation > 0.98 compared with PSA Delfia in 70 samples from subjects with BPH and prostate cancer (data not shown).

The relative response in the sample with PSA and ACT incubated for 2 h at 37°C were 99 ± 3% (mean ± s.d.) of the response of the control sample for the assays PSA10–PSA66, PSA10–PSA36, PSA66–PSA36 and PSA66–PSA42 using 25 μl of sample and 30 + 30 min incubation time. The PSA42–PSA29 assay showed a relative response of 84 ± 3% using 30 + 30 min incubation and 97 ± 2% using 1 + 1 h incubation (Table 2).

### Design of assays for determination of F-PSA

The MAbs of Group B could not be combined with each other in sandwich assays. However, combination of MAbs of Group B and Group A resulted in assays specific for F-PSA. In particular, combination of the Group B MAbs either as catching MAb or as detecting MAb together with MAb PSA66 of Group AIIa or PSA 36 MAb of Group AIIIb resulted in assays with high sensitivity. The specificity of these assays, i.e. cross-reaction between F-PSA and PSA–ACT, was << 1% determined either by response of purified PSA–ACT complex or determination of response in eluted fractions corresponding to PSA–ACT after separation of F-PSA and PSA–ACT by size exclusion chromatography on Sephacryl S-100. The immunoassays using PSA42 and/or PSA54 either as catching or detecting antibody in combination with the Group B Mabs did not give any dose–response curves, and a poor dose–response relationship was also seen with antibodies of group AIa and AIIIa. This is in agreement with the fact that the PSA 42 and PSA54 MAbs almost completely inhibited and that the AIa and AIIIa antibodies partly inhibited the binding of the group B MAbs. The results indicated that the epitopes of these antibodies may be located close to each other in the native PSA molecule.

**Table 2** Relative response of different PSA assays

| Assay | Samples % relative response[a] Control | Test |
|---|---|---|
| PSA10–PSA66[b] | 100 | 102 ± 1.5 |
| PSA10–PSA36 | 100 | 98 ± 2.1 |
| PSA66–PSA36 | 100 | 100 ± 1.1 |
| PSA66–PSA42 | 100 | 99 ± 1.4 |
| PSA42–PSA29, 30 + 30 min | 100 | 84 ± 2.5 |
| PSA42–PSA29, 60 + 60 min | 100 | 97 ± 0.9 |
| PSA10–PSA30 | 100 | 59 ± 1.4 |

[a]The signal in the control sample was assigned 100% and the signal in the test sample was given as a percentage of the signal in the control sample; [b]PSA10–PSA66 indicates catching MAb–tracer MAb. The control sample consisted of seminal plasma containing 100 μg of PSA in 2 ml of PBS and incubated 2 h at 37°C before analysis. The test sample consisted of seminal plasma containing 100 μg of PSA plus 500 μg of ACT in 2 ml of PBS and incubated for 2 h at 37°C before analysis. The samples were diluted 1: 200 before analysis in quadruplicates in the respective IFMA prototype assay. All assays used the following assay protocol unless otherwise stated in the table: 25 μl sample + 100 μl assay buffer, 30 min incubation; washing three times; 100 μl of Eu-labelled tracer (1 μg $ml^{-1}$) incubation 30 min; washing six times; 200 μl of enhancement solution and determination of Eu fluorescence in an Arcus fluorometer.

### Assays for determination of PSA–ACT

Using antibodies from Group A as catching MAbs and MAbs from group C as detecting MAbs, it was possible to design two-step sandwich assays for specific determination of the PSA–ACT complex without (<< 1%) cross-reactivity with F-PSA (data not shown).

### DISCUSSION

The existence of different molecular forms of PSA in serum and significant differences in the proportions of the serological forms of PSA between individuals (Lilja et al, 1991; Stenman et al, 1991; Christensson et al, 1993) and between benign and malignant prostate disease has further increased the importance of well-characterized immunological reagents for standardization of PSA immunoassays (Graves et al, 1990; Graves, 1993; Stamey et al, 1994) and for development of assays for specific determination of different forms of PSA.

In this paper, antigenic domains of PSA were characterized in order to find optimal pairs of MAbs to be used in immunoassays for determination of F-PSA and total PSA (i.e. F-PSA+PSA–ACT complex).

The epitope mapping indicated a complex pattern of eight to nine unrelated and partly overlapping antigenic domains in PSA. The complexity of the antigenic domains was further increased by the existence of several epitopes within each domain (e.g. see separation of Group AI into at least two subgroups, Figure 1A–C). In order to illustrate the possible relationship between the different antigenic domains, a hypothetical 'map' of the antigenic domains

 © Cancer Research Campaign 1997

**Figure 5** The group A MAb recognized epitopes exposed both in F-PSA and PSA–ACT, while the group B MAb recognized epitopes that were covered by the ACT and only exposed in F-PSA. The figure gives an illustration of the possible relation between the different domains recognized by the established MAbs of PSA was drawn (Figure 5). The Group A MAb recognizing epitopes exposed both in F-PSA and in PSA–ACT detected four unrelated domains (AI, AII, AIII and AV) that showed no or low inhibition between each other and three antigenic domains partly overlapped by the other domains.

One antigenic domain specific for F-PSA was detected which consisted of at least one continuous and two probably discontinuous epitopes. In general, there was a clear distinction between the antibodies that recognized exposed epitopes (i.e. epitopes that are detected both in F-PSA and PSA–ACT) and hidden epitopes (i.e. epitopes specific for F-PSA). However some antigenic domains may be located close to the 'F-PSA specific' domain as a partial inhibition of the F-PSA specific MAb was seen (e.g. group AIIIa, AV and VI). The close proximity of these epitopes and the F-PSA domain was also supported by the fact that these antibodies resulted in poor sandwich immunoassays with antibodies specific for F-PSA, and that immunoassays using these antibodies did not give equimolar assays for F-PSA and PSA–ACT during the experimental conditions used. Lövgren et al (1995) and Pettersson et al (1996) have characterized the epitopes of different PSA antibodies by determination of the dose–response of different antibody combinations (including the PSA10, PSA36, PSA30, PSA6, PSA19, PSA20 MAbs) with PSA, PSA–ACT, recombinant PSA and recombinant hK2. Their studies showed that none of the antibodies specific for F-PSA cross-reacted with hK2 and indicated also that the PSA10 MAb and PSA36 MAb were specific for PSA, i.e. without cross-reactivity with hK2. Preliminary in-house data confirm these results and suggest that the separation of the AI and AIII into subgroups would be dependent on difference in cross-reactivity with hK2, i.e. group AIb and AIIIb seem to be specific for PSA, while antibodies of group AIa and AIIIa may cross-react with hK2 (unpublished observation).

Chu et al (1989) recognized two overlapping antigenic domains using five MAbs, and Belanger et al, (1993) detected four antigenic domains using a large number of MAbs directed against PSA. The MAbs did not completely inhibit a polyclonal anti-PSA antiserum, in any of the studies, indicating that additional antigenic domains would be available. A complex pattern of overlapping and distinct determinants of PSA was revealed by studying all possible two-site immunoassays of 12 MAbs against PSA (Pettersson et al, 1995). In our study, mixing MAbs of different groups resulted in a step-wise increased inhibition of a polyclonal rabbit anti-PSA antiserum, which clearly indicated that antibodies of the different groups recognized different antigenic domains in PSA. The initial screening procedure used in this study with unlabelled PSA in solution may have exposed epitopes not exposed in PSA-coated on solid phases or in labelled PSA, which may explain the large number of different antigenic domains detected in this study compared with previous studies. All studies indicate a surprisingly complex epitope map of PSA, and further characterization of the reactivity with hK1, hK2 and different recombinant forms of PSA would be needed in order to define true PSA-specific antigenic domains.

Reduction of the five intra-chain S–S bonds in PSA and denaturation with SDS will completely destroy the native three-dimensional structure and also separate PSA into intact PSA and 'nicked' forms of PSA (i.e. PSA with internal cleavage of the amino acid sequence which in the native form is held together by the intra-chain S–S bonds). In this study, Western blot analysis of reduced and non-reduced PSA by SDS-PAGE was used to separate antibodies whose recognition of PSA was dependent only on the linear sequence of amino acids (i.e. true continuous epitopes) and those that were dependent on the three-dimensional structure of PSA. Non-reduced PSA was recognized in the Western blot analysis after SDS-PAGE by all MAbs, but there were clear differences in reactivity with reduced PSA between the MAbs. Reactivity with reduced PSA strongly suggested that the epitopes consisted of linear sequences of amino acids. The AIII MAbs (and polyclonal antibody anti-PSA) reacted also with fragments of PSA indicating that these antibodies also recognized 'nicked' forms of PSA. Those MAbs that only recognized non-reduced PSA may recognize discontinuous epitopes dependent on the three-dimensional structure of the PSA molecule for binding, but it is not possible to exclude the existence of continuous epitopes also within this group. Although both reduced and non-reduced PSA was recognized by some of the anti-PSA MAb, the reactivity was clearly lower with the reduced form of PSA. Thus, the native conformation of the PSA molecule was of major importance for the binding to PSA of all MAbs.

The reaction between PSA and $\alpha_2$-macroglobulin leads to total encapsulation of the PSA molecule, and the PSA–$\alpha_2$M complex is not detected in conventional immunoassays. Denaturation of the PSA–$\alpha_2$M complex with SDS leads to the formation of two subunits with an approximate molecular weight of 380 kDa and exposure of the PSA moiety; and PSA–$\alpha_2$M has been detected in human serum using SDS-PAGE (Zhou et al, 1993). Interestingly, there was no difference in the recognition of PSA–$\alpha_2$M in SDS-PAGE between MAbs recognizing epitopes exposed both in F-PSA and PSA–ACT and the MAbs recognizing epitopes exposed only in F-PSA.

The proteolytic activity of PSA against the S-2586 substrate was almost completely inhibited by most antibodies, and only the antibodies PSA66, PSA71, PSA67, PSA45 and PSA36 inhibited the proteolysis by less than 50%. It was expected that the antibodies recognizing epitopes specific for F-PSA would inhibit the proteolytic activity of PSA, but it was expected less that most antibodies against exposed epitopes inhibited the enzymatic activity of PSA. The results suggested that binding of anti-PSA MAb to PSA induced conformational changes leading to loss of enzymatic activity of PSA.

At present, there are three areas of main interest related to the design of PSA immunoassays: ultrasensitivity, equimolar response assays (i.e. assays with the same molar response for F-PSA and PSA–ACT) and assays for specific determination of the different serological forms of PSA. The novel luminometric and fluorometric detection methods and high-affinity antibodies make a lower limit of detection (LLD) < 0.005 μg $l^{-1}$ possible, but the clinical need for sensitivity of PSA assays indicates the need for

© Cancer Research Campaign 1997

further clinical studies (Vessella, 1993; Vessella and Lange 1993; Yu and Diamanidis, 1993; Prestigiacomo and Stamey, 1994). The detailed specificity of the immunoreagents is of great importance for determination of PSA in the ultrasensitive range to exclude determination of other members of the kallikrein gene family.

The terms equimolar and skewed-response assays have been introduced to describe the differences in molar response between F-PSA and PSA–ACT of immunoassays (Graves 1993). Differences in the molar response for F-PSA and PSA–ACT may partly explain the discordant results between assays based on monoclonal antibodies (mono–mono assays), polyclonal antibodies (poly–poly assays) or assays using both mono-and polyclonal antibodies (mono–poly assays). In poly–poly or mono–poly assays, non-equimolar response may be obtained because additional epitopes are available for the polyclonal antibodies in F-PSA compared with PSA–ACT thus leading to an overestimation of the F-PSA fraction. However, the problem of non-equimolar response is also evident in mono–mono assays, and diagnostic kits using mono–mono design with large differences in molar response between F-PSA and PSA–ACT are available. Reports have suggested that poly–mono assays may reflect the clinical course of prostate cancer more accurately than mono–mono assays (Bluestein et al, 1992) and may also be less sensitive towards interference from heterophilic antibodies than mono–mono assays (Slota et al, 1994). Thus, the proper choice of monoclonal antibodies is of major importance for the design of immunoassays giving equimolar response for F-PSA and PSA–ACT and maximum recognition of the different forms of PSA in serum.

The combination of the MAbs of Group A resulted in several sandwich assays with similar molar response for F-PSA and PSA–ACT. In particular the combination of MAbs of Group AIb, AII, AIII and AV resulted in highly sensitive assays (LLD << 0.01 $\mu g\ l^{-1}$ using 25 μl of sample) for determination of total PSA (i.e. F-PSA + PSA–ACT). The sandwich assays using PSA10–PSA66, PSA10–PSA36, PSA66–PSA36 and PSA66–PSA42 MAbs gave an equal response to the control sample containing only F-PSA and the test sample containing ≈ 60% F-PSA and ≈ 40% PSA–ACT, suggesting an equimolar response for F-PSA and PSA–ACT of these assays. The equimolarity of the assays were also supported by the identical dose–response and kinetics of purified F-PSA and PSA–ACT. The combination of PSA42–PSA29 showed a lower relative response in the test sample using 30 + 30 min incubation, suggesting a non-equimolar response using this assay configuration, while 60 + 60 min incubation showed almost the same response as in the control sample. Further studies indicated that the non-equimolar response of the PSA42–PSA29 assay was as a result of the use of PSA42 MAb as catching MAb. Thus, the proper choice of immunoreagents, orientation of the antibodies as well as the general assay design will influence the molar response between F-PSA and PSA–ACT of different mono–mono sandwich PSA assays.

The initial findings of lower proportions of F-PSA in prostate cancer than in BPH (Stenman et al, 1991; Christensson et al, 1993) and the improved discrimination between BPH and prostate cancer by determination of the ratio between F-PSA and total PSA or PSA–ACT has focused interest on immunoassays specific for F-PSA and/or PSA–ACT. In order to obtain optimal specificity of the F-PSA–total PSA ratio, it is essential that the assays used for determination of F-PSA show minimal cross-reactivity between F-PSA and PSA–ACT and that the total PSA assay gives an equimolar response for F-PSA and PSA–ACT. The F-PSA specific antibodies recognized the same or very similar antigenic domain and could thus not be combined in sandwich assays. To our knowledge, there are no F-PSA-specific antibodies available that can be combined in sandwich pairs, suggesting that the F-PSA-specific antigenic domain is relatively restricted. The combination of the F-PSA specific MAbs and MAbs of Group AIIa or AIIIb resulted in highly sensitive and specific assays for determination of F-PSA. The cross-reactivity of the preferred configurations was clearly < 1% (0.1–0.2%) suggesting that these assays would be suitable for further clinical evaluation of determination of F-PSA. Enzyme immunoassays for specific determination of F-PSA and total PSA have been developed using the PSA30, PSA10 and PSA66 MAbs (Nilsson et al, 1994) and used for determination of F-PSA and the ratio between F-PSA and total PSA in BPH, prostate cancer and healthy individuals (Nilsson et al, 1994*a*).

The MAb directed against the ACT portion of the PSA–ACT complex could be used as detecting MAb together with the catching MAb of group A for the development of sensitive and specific sequential sandwich assays for determination of PSA–ACT. Determination of the ratio between F-PSA and PSA–ACT instead of F-PSA–T-PSA ratio would theoretically further improve the discrimination between BPH and CAP. However, assays using anti-ACT antibodies as tracer may show severe background problems because of non-specific adsorption of cathepsin G and/or ACT to plastic surfaces, resulting in binding of the anti-ACT tracer; this has limited the practical usefulness of this assay design for specific determination of PSA–ACT (Leinonen et al, 1993). Therefore, our approach for the development of PSA–ACT-specific assays is to try to establish MAbs specific for the PSA–ACT complex without reactivity with active ACT and to use these antibodies for development of PSA–ACT-specific assays which should not give problems related to unspecific adsorption of an anti-ACT tracer.

At present, there are independent studies using different immunological reagents and patient materials (Nilsson et al, 1994*b*; Wang et al, 1994; Prestigiacomo and Stamey, 1995) which have confirmed the initial finding that specific determination of different serological forms of PSA may significantly improve the discrimination between benign and malignant prostate disease (Stenman et al, 1991; Christensson et al, 1993). However, the exact molecular differences and a biological explanation for the difference in PSA between BPH and prostate cancer has so far not been elucidated. Carefully characterized monoclonal antibodies directed against different antigenic domains of PSA may be useful not only for development of immunoassays for determination of different serological forms of PSA but also for the further immunological characterization of the different forms of PSA. The 'F-PSA' fraction may be heterogeneous containing different forms of enzymatically inactive PSA. Preliminary studies have suggested differences in the proteolytic activity of PSA in serum from healthy individuals and BPH compared with subjects with prostate cancer (Paus et al, 1995); this could explain the different proportions of F-PSA between BPH and CAP. The detailed biochemical and immunological characterization of PSA and/or PSA-related substances will be needed to further clarify the 'true' biochemical and molecular nature of the different serological forms of PSA.

### ACKNOWLEDGEMENT

The valuable discussions and comments by Dr Hans Lilja, University of Lund, Sweden, during the preparation of the manuscript is gratefully acknowledged.

© Cancer Research Campaign 1997

### ABBREVIATIONS

PSA, prostate-specific antigen; F-PSA, free PSA, i.e. PSA not in complex with any protease inhibitor; PSA–ACT, PSA in complex with $\alpha_1$-anti-chymotrypsin; PSA-a$_2$M, PSA in complex with $\alpha_2$-macroglobulin; kDa, kilodalton; SDS-PAGE, sodium dodecylsulphate polyacrylamide electrophoresis; Delfia, dissociation enhanced fluoroimmunoassay; ATCC, American Type Culture Collection; DMEM, Dulbecco's modified Eagle medium; MTP, microtitre plate; HRP, horseradish peroxidase; OPD, orthophenyl diamine; LLD, lower limit of detection; BPH, benign prostatic hyperplasia; CAP, cancer of the prostate; TTBS, tris-buffered saline containing Tween 20.

### REFERENCES

Akiyama K, Nakamura T, Iwanaga S and Hara M (1987) The chymotrypsin-like activity of human prostate-specific antigen, γ-seminoprotein. *FEBS Lett* **255:** 168–172

Belanger A, Rong PM, Liu S, Lavoi L and Labrie F (1993) Characterization and measurement of prostate-specific antigen using monoclonal antibodies. *Clin Invest Med* **16:** 409–414

Bluestein B, Zhou A, Tewari P, Comerci C, Schubert W and Larsen FL (1992) Multi-site clinical evaluation of an automated chemiluminescent immuno-assay for prostate-specific antigen (ACS™ PSA). *J Tumor Marker Oncol* **7:** 41–60

Christensson A (1993) *Prostate-Specific Antigen. Enzyme Activity and Reactions with Extracellular Protease Inhibitors* Thesis University of Lund

Christensson A, Laurell C-B and Lilja H (1990) Enzymatic activity of prostate-specific antigen and its reactions with extracellular serine proteinase inhibitors. *Eur J Biochem* **194:** 755–763

Christensson A, Björk T, Nilsson O, Dahlén U, Matikainen M-T, Cockett ATK, Abrahamsson PA and Lilja H (1993) Serum prostate specific antigen complexed to $\alpha_1$ antichymotrypsin as an indicator of prostate cancer. *J Urol* **150:** 100–105

Chu TM, Kawinski E, Hibi N, Croghan G, Wiley J, Killian CS and Corral D (1989) Prostate-specific antigenic domain of human prostate specific identified with monoclonal antibodies. *J Urol* **141:** 152–156

Graves HCB (1993) Standardization of immunoassays for prostate-specific antigen. A problem of prostate-specific antigen complexation or a problem of assay design? *Cancer* **72:** 3141–3144

Graves HCB, Wehner N and Stamey TA (1990) Comparison of a polyclonal and monoclonal immunoaasy for PSA: need for an international antigen standard. *J Urol* **144:** 1516–1522

Hemmilä I, Dakubu S, Mukkala VM, Siitari H and Lövgren T (1983) Europium as a label in time-resolved immunofluorometricc assays. *Anal Biochem* **137:** 335–340

Leinonen J, Lövgren T, Voranen T and Stenman U-H (1993) Double-label time-resolved immunoflurometric assay of prostate-specific antigen and its complex with $\alpha_1$-antichymotrypsin. *Clin Chem* **39:** 2098–2103

Lilja H (1993) Significance of different molecular forms of serum PSA *Urol Clin N Am* **20:** 681–686

Lilja H (1985) A kallikrein-like serine protease in prostatic fluid cleaves the predominant seminal vesicle protein. *J Clin Invest* **76:** 1899–1903

Lilja H, Oldbring J, Rannevik G and Laurell C-B (1987) Seminal vesicle-secreted proteins and their reactions during gelation and liquefaction of human semen. *J Clin Invest* **80:** 281–285

Lilja H, Christensson A, Dahlén U, Matikainen M-T, Nilsson O, Pettersson K and Lövgren T (1991) Prostate-specific antigen in serum occurs pre-dominantly in complex with $\alpha_1$-antichymotrypsin. *Clin Chem* **37:** 1618–1625

Lilja H, Cockett ATK and Abrahamsson P-A (1992) Prostate-specific antigen predominantly in complex with $\alpha_1$-antichymotrypsin *Cancer* **70:** 230–234

Lindolm L, Holmgren J, Svennerholm L, Fredman P, Nilsson O, Persson B, Myrvold H and Lagergård T (1983) Monoclonal antibodies against gastro-intestinal tumour-associated antigens isolated as monosialo-gangliosides. *Int Archs Allergy Appl Immunol* **71:** 178–181

Lundwall Å (1989) Characterization of the gene for prostate-specific antigen, a human glandular kallikrein. *Biochem Biophys Res Commun* **161:** 1151–1159

Lundwall Å and Lilja H (1987) Molecular cloning of human prostate-specific antigen cDNA. *FEBS Lett* **214:** 317–322

Lövgren J, Piironen T, Övermo C, Dowell B, Karp M, Pettersson K, Lilja H and Lundvall Å (1995) Production of recombinant PSA and HK2 and analysis of their immunological cross-reactivity. *Biochem Biophys Res Comm* **213:** 888–895

Nilsson O, Dahlén U, Grundström B, Karlsson B and Nilsson K (1994*a*) Development of immunoassays specific for the different serological forms of PSA. In *Current Tumor Diagnosis: Applications, Clinical Relevance, Research, Trends* Klapdor R (ed), pp. 289–296 W Zuckswerdt: München

Nilsson O, Dahlén U and Karlsson B (1994*b*) Free and total PSA in healthy subjects and in patients with benign and malignant prostatic disease. In *Basic Research and Clinical Application in Human Tumor Immunology and Molecular Biology*, Proceedings of the XXIInd Meeting of ISOBM, Groningen 1994, p. 319

Oesterling JE (1991) Prostate specific antigen: A critical assessment of the most useful tumor marker for adenocarcinoma of the prostate. *J Urol* **145:** 907–923

Paus E, Fossa SD, Nilsson O, ERI M and Börmer O (1995) The ratio of free to total PSA may be influenced by handling of serum samples. 8th International Hamburg Symposium On Tumor Markers

Pettersson K, Piironen T, Seppälä M, Liukkonen L, Christensson A, Matikainen MT, Suonpää M, Lövgren T and Lilja H (1995) Free and complexed prostate-specific antigen (PSA): in vitro stability, epitope map, and development of immunofluorometric assays for specific and sensitive detection of F-PSA and PSAa1-antichymotrypsin complex. *Clin Chem* **41:** 1480–1488

Pettersson K, Piironen T, Karp M, Lövgren T, Dowell B, Lövgren J and Lilja H (1996) Novel, specific and sensitive immunoassay for hK2 measures low concentrations of serum hK2 compared to PSA. *J Urol* **155** (suppl.) 695A

Prestigiacomo AF and Stamey TA (1994) A comparison of 4 ultrasensitive prostate specific antigen assays for early detection of residual cancer after radical prostatectomy. *J Urol* **152:** 1515–1519

Prestigiacomo AF and Stamey TA (1995) Clinical usefulness of free and complexed PSA. *Scand J Clin Lab Invest* (suppl.) **221:** 32–34

Riegman PH, Vliestra RJ, Van Der Korput J, Romijn J and Trapman J (1989) Characterization of the prostate-specific antigen gene: a novel human kallikrein-like gene. *Biochem Biophys Res Comm* **159:** 95–102

Schedlich LJ, Bennetts B and Morris BJ (1987) Primary structure of a human glandular kallikrein gene. *DNA* **6:** 429–437

Slota J, Prine B, Magic S, Dowell B and Huhn O (1994) Detection of a circulating immune complex of PSA and IgG in a patient with benign prostatic hyperplasia and hypergammaglobulinemia In *Basic Research and Clinical Application in Human Tumor Immunology and Molecular Biology*, Proceedings of the XXIInd Meeting of ISOBM, Groningen 1994, p 312

Stamey TA, Prestigiacomo AF and Chen Z (1994) Standardization of immunoassays for prostate-specific antigen: a different view based on experimental observation. *Cancer* **74:** 1662–1666

Stenman UH, Leinonen J, Alfthan H, Ranniko S, Tuhkanen K and Alfthan O (1991) A complex between prostate specific antigen and $\alpha_1$-antichymotrypsin is the major form of prostate-specific antigen in serum of patients with prostatic cancer: assays of the complex improves clinical sensitivity for cancer. *Cancer Res* **51:** 222–226

Vessella RL (1993) Trends in immunoassays of prostate-specific antigen: serum complexes and ultrasensitivity. *Clin Chem* **39:** 2035–2039

Vessella RL and Lange PH (1993) Issues in the assessment of PSA immunoassays. *Urol Clin North Am* **20:** 607–619

Wang MC, Valenzuela LA, Murphy GP and Chu TM (1979) Purification of a human prostate specific antigen. *Invest Urol* **17:** 159–163

Wang TJ, Hill T, Sokoloff R, Frankenne F, Wolfert R and Rittenhouse H (1994) Monoclonal antibody sandwich immunoassay to quantitate F-PSA in benign prostate hyperplasia and prostate cancer. In *Basic Research and Clinical Application in Human Tumor Immunology and Molecular Biology*, Proceedings of the XXIInd Meeting of ISOBM, Groningen 1994, p 317

Watt KWK, Lee P-J, M'timkulu T, Chan W-P and Loor R (1986) Human prostate-specific antigen: structural and functional similarity with serine proteases. *Proc Natl Acad Sci USA* **83:** 3166–3170

Wood WG, Van Der Sloot E and Böhle A (1991) The establishment and evaluation of luminiescent-labeled immunometric assays for prostate-specific antigen-$\alpha_1$-antichymotrypsin complexes in serum. *Eur J Clin Chem Clin Biochem* **39:** 787–794

Yu H and Diamanidis EP (1993) Ultrasensitive time-resolved immunofluorometric assay of prostate-specific antigen in serum and preliminary clinical studies. *Clin Chem* **39:** 2108–2114

Zhou A, Tewari PC, Bluestein BI, Caldwell GW, Larsen FL (1993) Multiple forms of prostate-specific antigen in serum: differences in immuno-recognition by monoclonal and polyclonal assays. *Clin Chem* **39:** 2483–2491

© Cancer Research Campaign 1997